# *Drosophila* Pif1A is essential for spermatogenesis and is the homolog of human CCDC157, a gene associated with idiopathic NOA

**DOI:** 10.1038/s41419-019-1398-3

**Published:** 2019-02-11

**Authors:** Xin Yuan, Huimei Zheng, Yang Su, Pengfei Guo, Xiao Zhang, Qiang Zhao, Wanzhong Ge, Chen Li, Yongmei Xi, Xiaohang Yang

**Affiliations:** 1 Division of Human Reproduction and Developmental Genetics, the Women’s Hospital, Zhejiang University School of Medicine, Yuhangtang Road 866, 310012 Hangzhou, Zhejiang China; 2 Institute of Genetics and Department of Genetics, Zhejiang University School of Medicine, Yuhangtang Road 866, 310012 Hangzhou, Zhejiang China; 3 Joint Institute of Genetics and Genomic Medicine between Zhejiang University and University of Toronto, Zhejiang University, Hangzhou, Zhejiang China

## Abstract

The dynamic process of spermatogenesis shows little variation between invertebrate models such as *Drosophila*, and vertebrate models such as mice and rats. In each case, germ stem cells undergo mitotic division to proliferate and then continue, via meiosis, through various stages of elongation and individualization from spermatogonia to spermatid to finally to form mature sperm. Mature sperm are then stored in the seminal vesicles for fertilization. Errors in any of these stages can lead to male infertility. Here, we identify that *Drosophila* Pif1A acts as a key regulator for sperm individualization. Loss of Pif1A leads to male sterility associated with irregular individualization complex and empty seminal vesicles without mature sperm. Pif1A is highly expressed in the testes of mated male adult flies and the Pif1A protein is expressed at a higher level in male than in female flies. Pif1A is homologous to mammalian coiled-coil domain-containing protein 157 (CCDC157), which is also enriched in the testes of humans and mice. Human CCDC157, with unknown function, was identified to be downregulated in men with idiopathic non-obstructive azoospermia (NOA). We map the function of *Drosophila* Pif1A during spermatogenesis, showing that Pif1A is essential for spermatide individualization and involved in the regulation of the lipid metabolism genes. Our findings might be applicable for studying the function of CCDC157 in spermatogenesis and other aspects of human male fertility.

## Introduction

Spermatogenesis is a highly conserved process, not differing appreciably between any animal model, from *Drosophila* in insects to mice and rats in mammals^1^. Each developmental stage is precisely controlled by intrinsic signals. Human male infertility results from abnormal spermatogenesis and is mostly due to chromosomal alterations, Y chromosome microdeletions, and related gene mutations^2^. For many of these, the detailed mechanisms are often technically inaccessible and remain obscure. In *Drosophila*, the testes are easy to dissect and the morphological changes during each stage of spermatogenesis can be easily visualized^3^. *Drosophila* is, therefore, well established as an excellent model for the study of spermatogenesis^4^. Recently, a large-scale RNAi screen in *Drosophila* testes has been conducted to analyze the genes required for germline stem cell (GSC) maintenance or differentiation^5^. Many of these genes were noted as those involved in key steps of protein synthesis and degradation relating to GSC homeostasis^5–7^, while others were identified as key regulators of the dynamic process of sperm morphogenesis^8,9^. However, the molecular links between different stages of spermatogenesis remains largely unidentified. The combination of various genetic techniques applied to *Drosophila*, such as CRISPR/Cas9^10^ and the Gal4/ UAS transgenic system^11^, have enabled the convenient mapping and prediction of functional genes which facilitate, for example, the identification of genes that might cause infertility due to deficiencies in a specific developmental stages or in certain type of cells.

To gain insight into the molecular basis for male sterility, we made use of CRISPR/Cas9 to produce mutations of the genes shown to be highly expressed in *Drosophila* testis. A gene of unknown function, PFTAIRE interacting factor 1A (*Pif1A*)^12^, was of particular interest. The female mutants of *Pif1A* displayed normal fertility but the males were infertile. *Pif1A* is the only homolog of the mammalian coiled-coil domain-containing protein 157 (CCDC157) family (www.pantherdb.org). Pif1A has been noted to exist at a high transcriptional expression level in the testes of mated male flies (www.flybase.org) and the Pif1A protein is expressed at a higher level in male than in female flies^12^. Notably, CCDC157 has also been observed as highly expressed in the testes of humans and mice, and to be downregulated in men with idiopathic non-obstructive azoospermia (NOA)^13,14^. Here, we map the function of Pif1A during *Drosophila* spermatogenesis, which might be informative for human CCDC157.

Male *Drosophila* adults contain a pair of testes, each testis is a coiled tube with a closed apical end and a basal end that connects to the seminal vesicle. At the apical end there are approximately 8–12 GSCs. Spermatogenesis takes place within individual units known as cysts^15^, where GSCs divide asymmetrically in the cysts to give rise to spermatogonial cells. Each GSC is flanked by two somatic cyst stem cells (CySCs) that eventually differentiate into a head cyst cell and a tail cyst cell, analogous to mammalian Sertoli cells^16,17^. Spermatogonia then go through four mitotic divisions, generating 16 primary spermatocytes that further undergo two meiotic divisions to yield a group of 64 syncytial haploid spermatids. Round spermatids are inter-connected by abundant cytoplasmic bridges^15,18^. During post-meiotic spermatid differentiation, syncytial cysts of 64 haploid spermatids undergo synchronous differentiation. Numerous changes occur at a subcellular level including formation of flagellar axonemes and acrosomes, remodeling of mitochondria and nuclei, and the polarization of elongating cysts and the plasma membrane^6^. The fully elongated syncytium of 64 spermatids undergoes a membrane remodeling process known as individualization^18,19^.

Individualization begins with the formation of investment cones around each of the 64 needle-shaped nuclei^16,18^. These investment cones assemble into a macroscopic structure referred to as the individualization complex (IC), from which the cyst membrane is remodeled and intercellular bridges are resolved to encase each sperm cell in its own plasma membrane^18,19^. The IC moves processively from the heads to the tips of the tails along the spermatid bundle^20^. During this process unneeded organelles, mitochondrial DNA, and cytoplasmic components are stripped away, forming the observable dilation of the cyst known as the “cystic bulge”. The cystic bulge is then detached at the tip of the tail where it becomes known as the “waste bag”^21^.

Individualization results in the formation of individual sperm. The structure of the actin cones and their cohort movement is critical for spermatid individualization. Cytoskeletal regulators, such as myosin V, myosin VI, cortactin, and Arp2/3 complex, have been identified to influence the formation of actin cones and the synchronous movement of the IC^22,23^. In myosin V mutants, fewer actin cones were able to form^24^. Myosin VI acts to stabilize the actin cones and the Arp2/3 complex is required for the formation of the actin meshwork^25–27^. Cortactin co-localizes with Arp2/3 complex and myosin VI at the leading edge of the actin cone during IC movement^28^.

The individualization process has also been defined as a caspase-dependent apoptosis-like event, requiring functional proteasomes and membrane trafficking^16,21^. Multiple *Drosophila* caspases and caspase regulators participate in this process. Mutation of any of these genes leads to individualization defects^9^. During complex membrane remodeling, lipids including phospholipids, sphingolipid and cholesterol, act as essential membrane structural components and also play critical roles. Lipid regulators including *Drosophila nessy (nes)*, oysgedart (oys)*, noa*, oxysterol binding protein (*osbp*), *fan, npc1* have been identified^29–32^, although the role of most of their protein products remains obscure. The mutation of *osbp*, for example, leads to abnormal sterols trafficking and distribution and in corresponding male infertility^30^. The regulation mechanism of OSBP remains unclear.

In this study, we have identified that *Pif1A* is essential for spermatid individualization and might act to regulate lipid metabolism genes during spermatogenesis. Loss of Pif1A results in the downregulation of several lipid regulators and in a phenotype that genetically mirrors that of the loss of *osbp*. By dissecting the testes and conducting immunofluorescence staining, we found that *Pif1A* mutants showed normal GSCs divisions and spermatid elongation but displayed disorganized IC and irregular IC movement. Correspondingly, no mature sperm could be observed in their seminal vesicles. The duplication of the *Pif1A* genome could partially rescue this phenotype. We show that *Pif1A* plays a critical role during spermatogenesis and thus is essential for male fertility. Our findings might be applicable for studying the function of CCDC157 in spermatogenesis and in other aspects of human male fertility.

## Results

### Identification of Pif1A, homologous to human CCDC157, as a novel regulator for male fertility

**Fig. 1:**
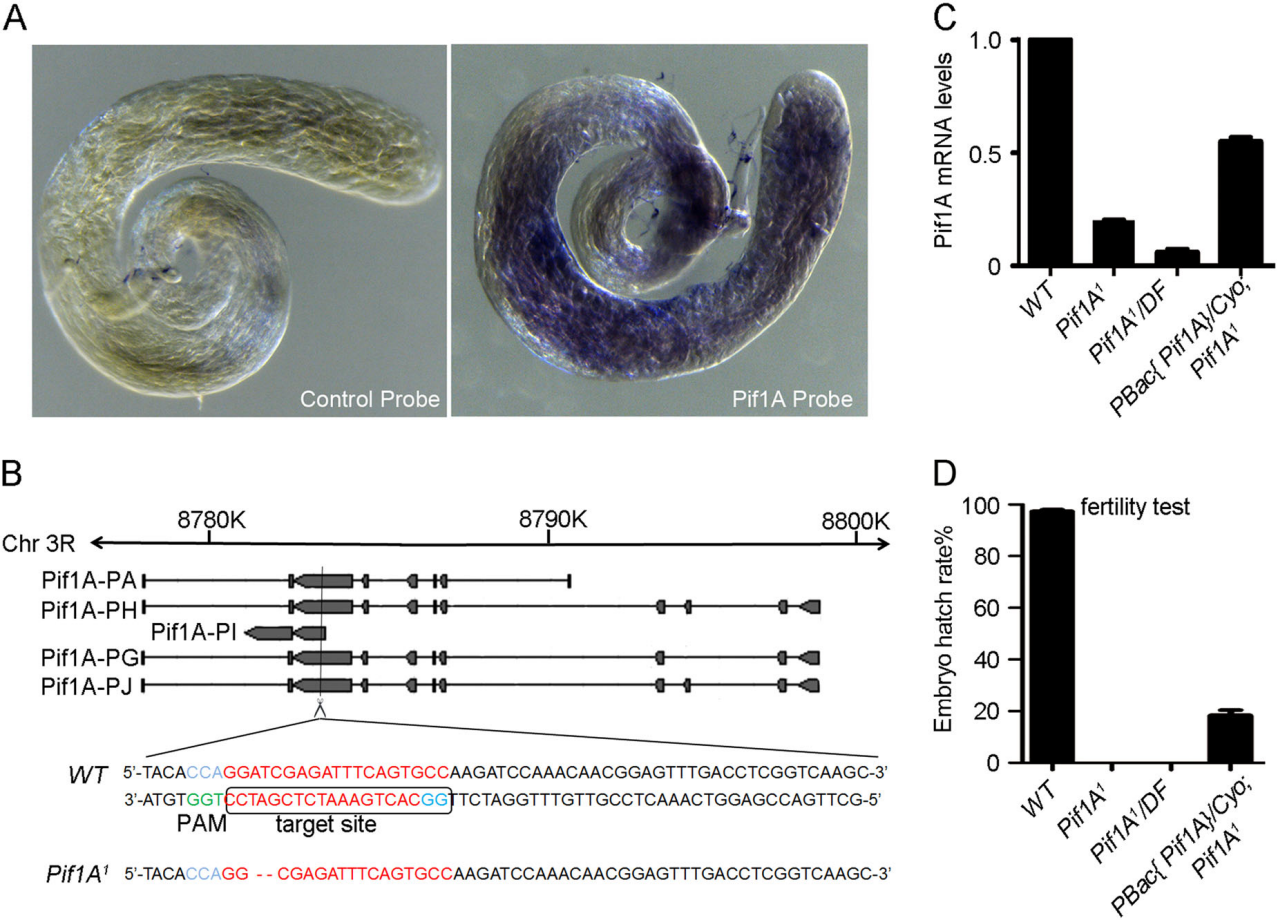
Loss-of-function of *Pif1A* results in male-sterile. **a** Whole-mount in situ hybridization for control probe (left) and Pif1A probe (right) on testis of adult flies. **b** Schematic representation of the *Pif1A* locus. *Pif1A* is located on chromosome 3R at 85B1–85B1 on the cytogenetic map. Filled tetragonums indicate exons; the straight lines between filled tetragonums indicate introns. *Pif1A*^*1*^ is a frame shift mutant allele with 2 bp deletion. **c** Real-time PCR analysis of *Pif1A* transcriptional levels of wild-type animals, *Pif1A*^*1*^ mutants, *Pif1A*^*1*^ over deficiency (*Pif1A*^*1*^/DF) animals and the duplication of *Pif1A* genomic DNA in the background of *Pif1A*^*1*^ (Pif1A-GFP.FPTB/Cyo;*Pif1A*^*1*^/*Pif1A*^*1*^). **d** Male fertility test. *y*-axis represented embryo hatch rate. The male fertility was zero in *Pif1A*^*1*^ mutants and *Pif1A*^*1*^/DF animals and partially restored in the duplication of *Pif1A* genomic DNA in the background of *Pif1A*^*1*^ animals

To identify novel regulators in spermatogenesis, using CRISPR/Cas9, we generated a series of mutants of the various genes that had high transcript expression levels in the testis of adult male (www.flybase.org). We focused on *Pif1A* and identified that one of the mutations, *Pif1A*^1^, resulted in compromised male fertility but left female fertility unaffected. Expression of *Pif1A* mRNA in WT adult testis is clarified by whole-mount in situ hybridization (Fig. 1a). The gRNA for *Pif1A* was designed to recognize a 19-nt target sequence in a position where it always plays as an exon role among five transcripts and acts to direct Cas9-mediated cleavage of both DNA strands within the target site. *Pif1A*^1^ is a frame shift nonsense mutant allele with 2 bp deletion (Fig. 1b) leading to an earlier stop codon 12 amino acids from the deletion position. We analyzed the transcription levels of *Pif1A*^*1*^ in homozygous adult flies and *trans* heterozygous adult animals from the *Pif1A*^*1*^ allele and a deficiency line uncovering *Pif1A* (*Pif1A*^*1*^*/DF*) by quantitative PCR (qPCR). The mRNA levels were significantly reduced in *Pif1A*^*1*^ and *Pif1A*^*1*^*/DF* male animals, indicating *Pif1A*^*1*^ is a loss-of-function mutation (Fig. 1c). This is the first loss of function allele for *Pif1A*, in contrast to the numerous other alleles listed in Flybase. Fertility test showed that WT females crossed with *Pif1A*^*1*^ or *Pif1A*^*1*^*/DF* males laid similar number of eggs as did those crossed with the WT males (Table S1), but the percentage of hatched embryos derived from *Pif1A* mutant males was zero (Fig. 1d). In addition, we carried out a genomic rescue experiment through a transgenic fly BL42670 (www.flybase.org). The duplication of the *Pif1A* genome was able to partially restore the male reproductive capacity (Fig. 1d).

**Fig. 2:**
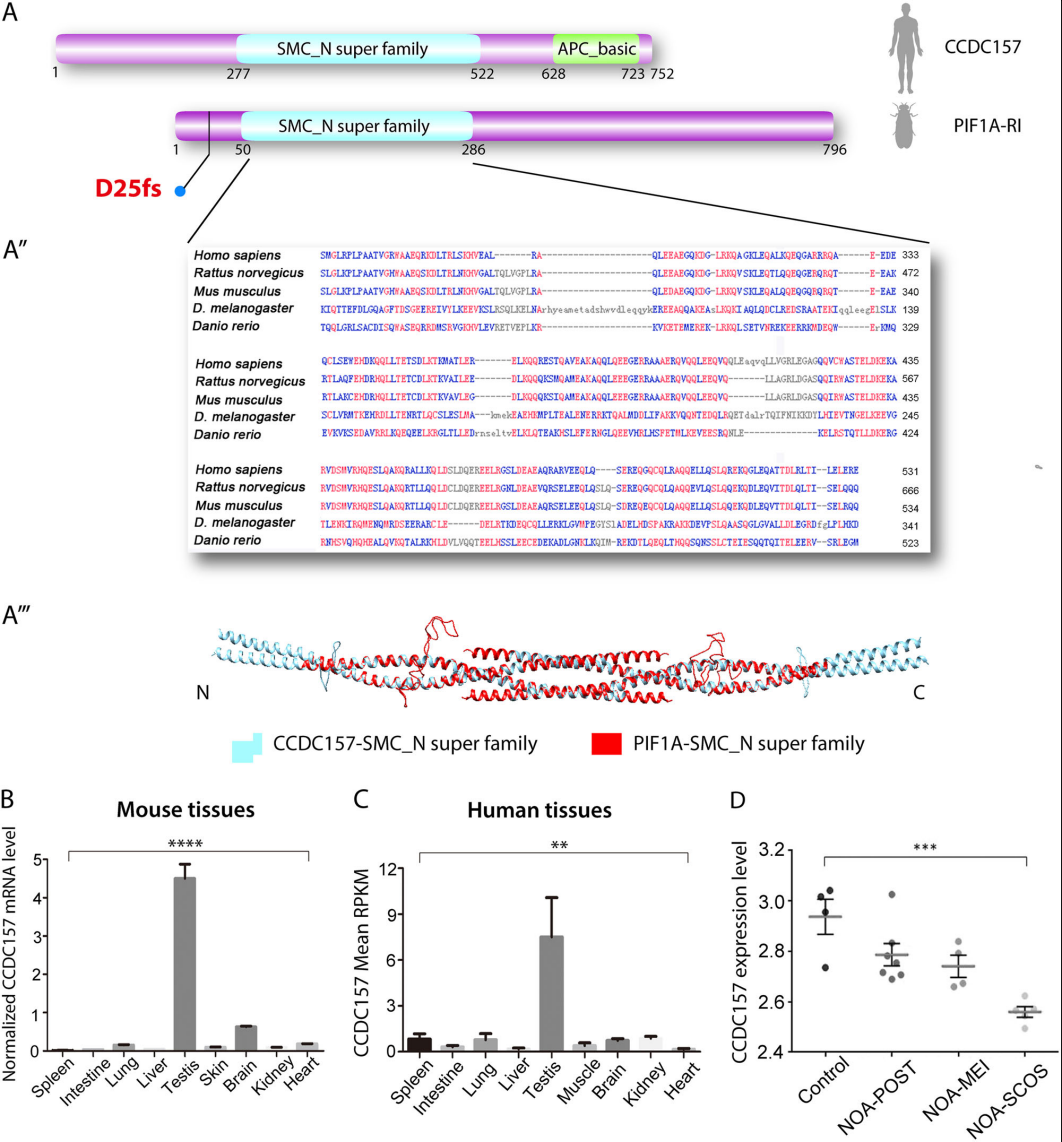
*Pif1A* homolog, CCDC157, is enriched in mammalian testis. **a** Schematic diagrams of human CCDC157 and *Drosophila* Pif1A-RI protein domain structure, blue color shows the SMC_N super family domain and all the five transcripts of *Pif1A* contained this domain; D25fs means that the 2 bp deletion mutation is at the 25th phenylalanine and later the frame shift generated the 12 amino acids followed by a stop code. **aʹ** Multiple sequence alignment of the SMC_N super family domains from *Drosophila melanogaster* Pif1A (fourth line) with *Homo sapiens* CCDC157 (top line), *Rattus norvegicus* CCDC157 (second line), *Mus musculus* CCDC157 (third line), and *Danio rerio* CCDC157 (bottom line). Amino acid sequences alignment columns with no gaps are colored in blue or red. The red color indicates at least three species are identical and blue indicates similar amino acids. **a**ʹʹ The superimposed 3D structure of the SMC_N super family domains from human CCDC157 (blue) and *Drosophila* Pif1A-RI (red) protein. SMC_N super family: SMC N terminal domain; APC_basic: APC basic domain. **b** Real-time PCR analysis of CCDC157 in various mouse tissues. The CCDC157 transcript was highly enriched in the mouse testis compared with the other tissues. **c** A quantitative transcriptomics analysis of human CCDC157. **d** CCDC157 mRNA expression levels in 16 patients with idiopathic non-obstructive azoospermia (NOA) compared with four men with normal spermatogenesis. The data was retrieved from Gene Expression Omnibus (GEO) GSE45887, where samples are obtained by the testicular biopsy specimens. ****P < 0.0001; ***P < 0.01; **P < 0.05; NOA, non-obstructive azoospermia; NOA-POST, post-meiotic arrest; NOA-MEI, meiotic arrest; NOA-SCOS, Sertoli-cell-only syndrome

Sequence analysis identified that *Drosophila Pif1A* is homologous to the mammalian CCDC157, with conserved SMC_N super family domain (Fig. 2a, aʹ, aʹʹ). The Pif1A protein has a 20% identity and 34% similarity to human CCDC157 and the predicted three-dimensional (3D) structure of Pif1A is similar to that of human CCDC157 (Fig. 2aʹʹʹ). We measured the mRNA levels of CCDC157 in various mouse tissues. The CCDC157 transcript was highly enriched in testes compared with the other tissues (Fig. 2b). We then performed a quantitative transcriptomics analysis through open databases^33^ and found that human CCDC157 was also highly enriched in the testes (Fig. 2c). The expression levels of CCDC157 in men with normal spermatogenesis were significantly higher than the patients with idiopathic NOA^13^(Fig. 2d). We further analyzed recently published single-cell RNA-sequencing data (GEO:GSE106487) underlying a hierarchical model of human testicular cells^14^. In addition to their findings on several stage-specific marker genes of testicular germ cells, such as HMGA1, PIWIL4, TEX29, SCML1, and CCDC112^14^, our analysis highlights that CCDC157 is also highly expressed in spermatid stages but rarely expressed in spermatogonia and spermatocyte stages (Fig. S1A). Totally, 2854 individual testicular cells from donors with normal spermatogenesis showed significant higher expression of CCDC157 than 174 testicular cells from one NOA donor (Fig. S1B).

### Pif1A mutants displayed normal GSC divisions and spermatid elongation, but an empty seminal vesicle

**Fig. 3:**
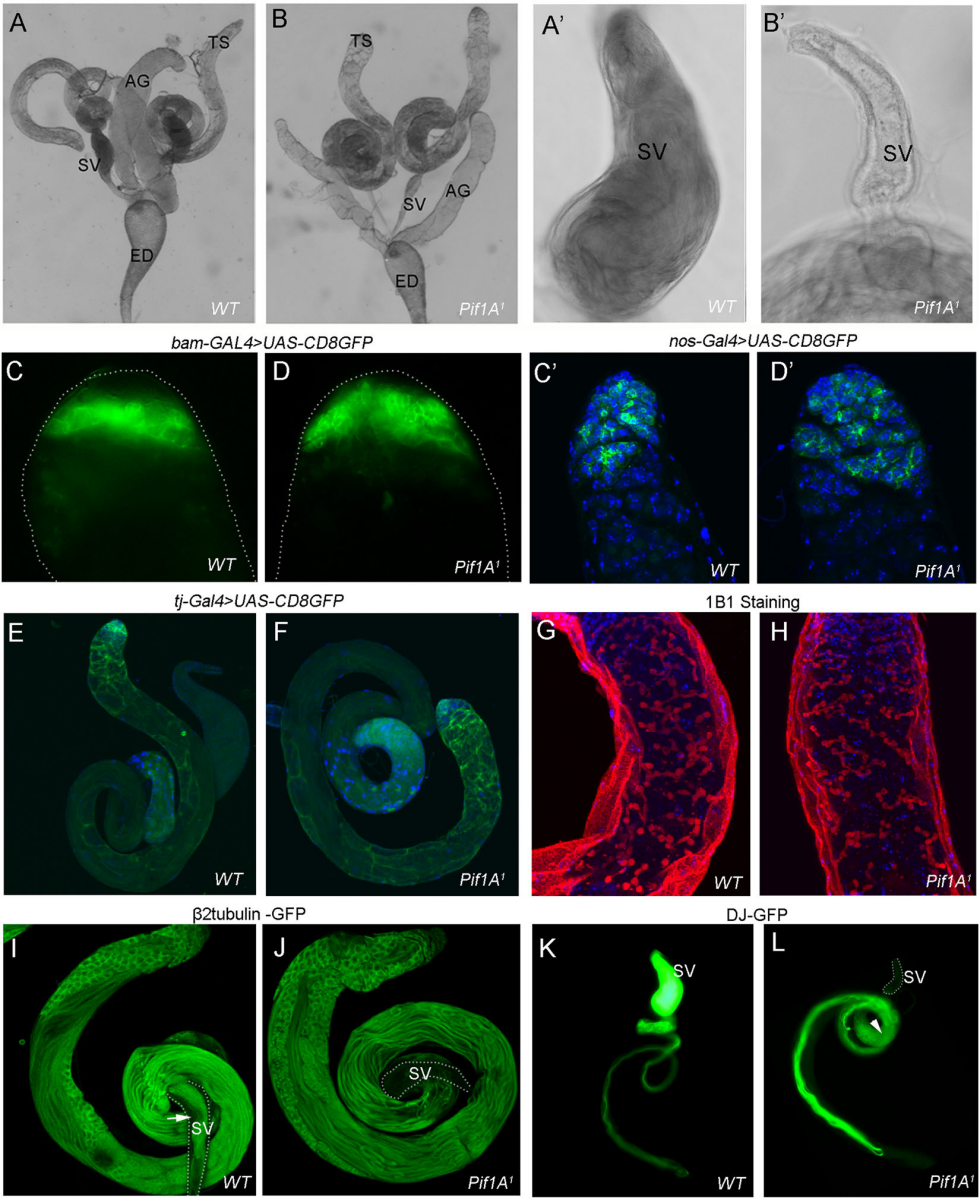
Germ cell divisions and spermatid elongation in *Pif1A*^*1*^ mutant seems unaffected. Images of dissected male reproductive system from wild-type animals (**a**) and *Pif1A*^*1*^ mutants (**b**); TS, testis; SV, seminal vesicle; AG, accessory gland; ED, ejaculatory duct. The magnified image of SV from wild-type animal (**aʹ**) was obviously larger than that of *Pif1A*^*1*^ mutant, which was empty (**bʹ**). Fluorescent images of the testes from control animals (bam-GAL4 > UAS-CD8GFP) (**c**) and *Pif1A*^*1*^mutants (bam-GAL4 > UAS-CD8GFP; *Pif1A*^*1*^/*Pif1A*^*1*^) (**d**) showed that spermatogonia had divided normally. Confocal images of the testes from control animals (nos-GAL4 > UAS-CD8GFP) (**cʹ**) and *Pif1A*^*1*^ mutants (nos-GAL4 > UAS-CD8GFP; *Pif1A*^*1*^/*Pif1A*^*1*^) (**dʹ**) indicated that early germ cells were not affected in *Pif1A*^*1*^mutant (Green;GFP, blue; DAPI). Images of the testes from control animals (tj-Gal4 > UAS-CD8GFP) and *Pif1A*^*1*^ mutants (tj-Gal4 > UAS-CD8GFP; *Pif1A*^*1*^/*Pif1A*^*1*^) (**f**) suggested that CySCs and cyst cells were also not affected in *Pif1A*^*1*^ mutants. 1B1 staining representing fusomes showed similar patterns in the testes of wild-type animals (**g**) and *Pif1A*^*1*^ mutants (**h**). The testis-specific β2tubulin promoter drives GFP shows that elongated spermatid bundles existing in both wild-type (**i**) and *Pif1A*^*1*^mutant (**j**), and seminal vesicles containing stored mature sperm (**i**, arrow) in wild-type animals but not in the *Pif1A*^*1*^ mutants. Elongated sperm tails, marked with Don Juan-GFP (green), fill the seminal vesicle in wild-type testes (**k**). In the *Pif1A*^*1*^ mutants, their elongated sperm tails are also shown in the testis but the seminal vesicle is empty (**l**)

We firstly examined the gonads of homozygous *Pif1A*^*1*^ adult fly. The morphology of the reproductive system, including accessory gland and ejaculatory duct, seemed normal in *Pif1A*^*1*^ compared to those of control animals (Fig. 3a, b). However, the seminal vesicle (SV) from the *Pif1A*^*1*^ male appeared completely empty and much smaller (Fig. 3aʹ, bʹ). Squashes of SVs confirmed the presence of motile sperm in the control animals as contrasted to their complete absence in *Pif1A*^*1*^ mutants.

We then examined the spermatogenesis processes, beginning with the evaluation of germ cell division. We constructed nos-GAL4, mainly expressed in early germ cells including GSCs but with faded expression in spermatocytes^34^, driving UAS-CD8GFP in *Pif1A* mutant background. By dissecting the gonad and analyzing GFP patterns, no obvious differences were found between the mutants and control animals (Fig. 3cʹ, dʹ). Similarly, the GFP patterns drove by bam-GAL4, mainly expressed in spermatogonia, or tj-Gal4, expressed in CySCs and cyst cells^5^, also appeared to be unaffected in *Pif1A* mutants (Fig. 3c–f). The *Pif1A*^*1*^ testes also showed normal intercellular connections^35^ (Fig. 3g, h). The primary spermatocyte cysts at the 16-cell stage and spermatid cysts at the onion stage (64-cell) are identical to those in the WT (Fig. S3A). These observations suggest that mutation of *Pif1A* has no effect on germ cell divisions.

We next explored the spermatid elongation by constructing an βTub85D-GFP as a spermatocyte-specific marker^36^, and a Don Juan-GFP, a marker for late spermiogenesis^33,37,38^ into the *Pif1A*^*1*^ background animals. The Don Juan protein is produced in the giant sperm tail’s mitochondria and peaks once the nuclei have condensed and persists into mature sperm, but is not found in spermatids that are still undergoing elongation^33,37,38^. Observing GFP markers, elongated sperm tails showed up as equally normal in both WT and *Pif1A*^*1*^ animals (Fig. 3i, l). However, the fluorescence was not detectable in the seminal vesicles of *Pif1A*^*1*^ mutants (Fig. 3j, l), but was present in that of WT animals (Fig. 3i, k). These results indicate that mutation of *Pif1A* may leads to a post-meiotic defect after spermatid elongation.

### Loss of Pif1A results in lack of cystic bulges and waste bags

**Fig. 4:**
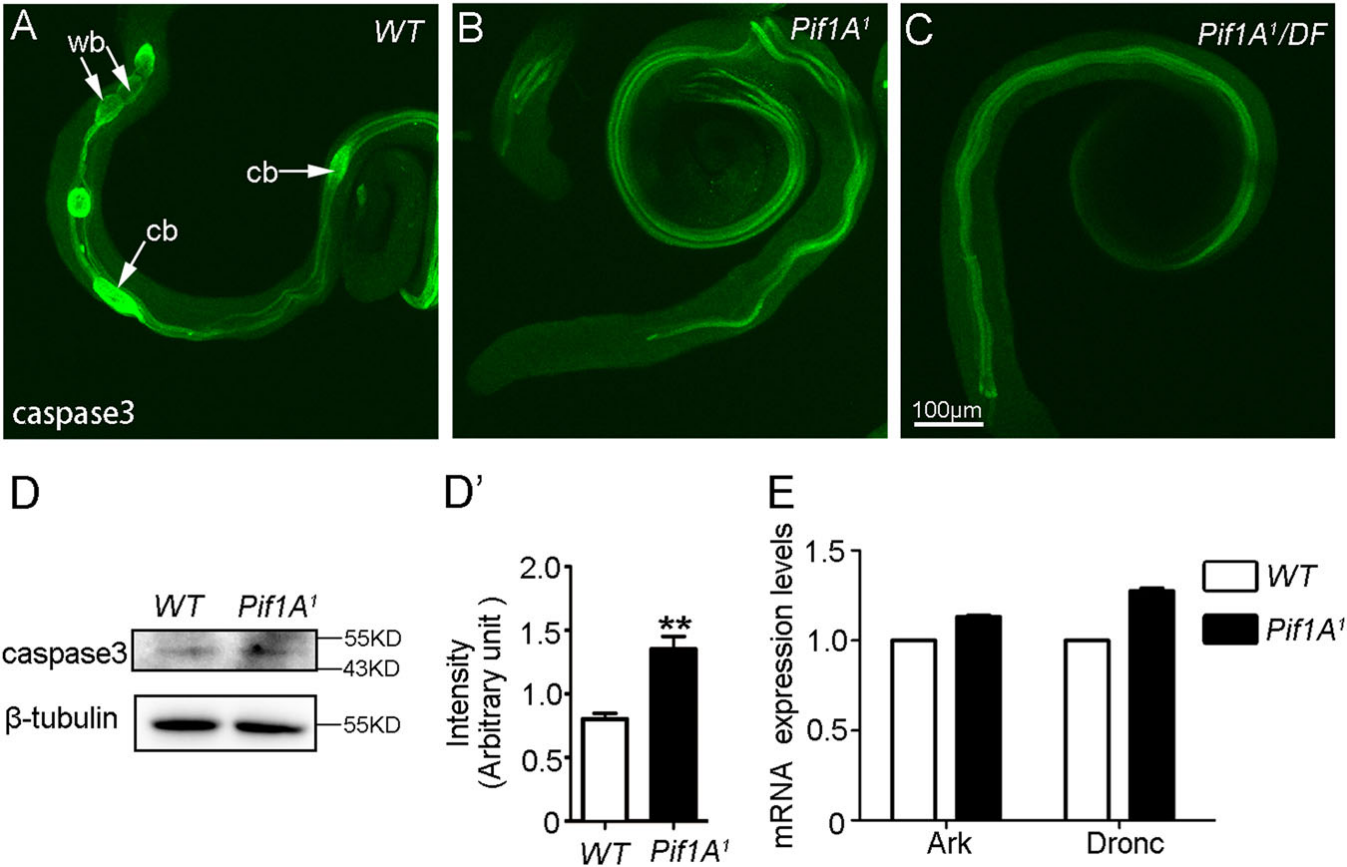
Loss-of-function of *Pif1A* leads to lacking of cystic bulges and waste bags. **a**–**c** Testes of different genotypes were visualized with antibodies specific for caspase 3 (green). **a** Wild-type testis. Caspase 3 was present in multiple elongated cysts. Cystic bulges (cb) and waste bags (wb) are indicated by arrows; **b**, **c** Testes from *Pif1A*^*1*^ homozygote mutants and *Pif1A*^*1*^/DF heterozygotes, respectively; cystic bulges and waste bags were hard to find. **d** Western blot detected by anti-caspase 3 and anti-tubulin antisera on lysates of testes from wild-type animals and *Pif1A*^*1*^ mutants. **d'** Quantification analyses of Casepase 3 in fig. 4**d**. **, p<0.01, Mann-whitney test, Mann-whitney test. **e** Real-time PCR analysis of *Ark* and *Dronc* transcriptional levels in the testes of wild-type animals and *Pif1A*^*1*^ mutants

The final stage of spermatid differentiation involves the removal of bulk cytoplasm. The DrICE is cleaved and activated throughout the length of elongated spermatids^9^. In WT testis, the elongated spermatids, cystic bulges (CBs) and waste bags (WBs) could be visualized with antibodies specific for activated DrICE (Fig. 4a). In *Pif1A*^*1*^ mutants, no CBs or WBs appeared, although DrICE-positive signals were partially detectable (Fig. 4b, c). We then extracted the protein and total RNA from the testes of *Pif1A*^*1*^ and WT animals. Western blot showed that the levels of active DrICE were not decreased (Fig. 4d, dʹ) and the transcript levels of two other genes related to caspase activity, *Ark* and *Dronc* were also not reduced in the mutants (Fig. 4e). These observations demonstrate that the lack of CBs and WBs in the testes of *Pif1A* mutants is not due to downregulated caspase activity.

### Pif1A is required for the spermatid individualization process

**Fig. 5:**
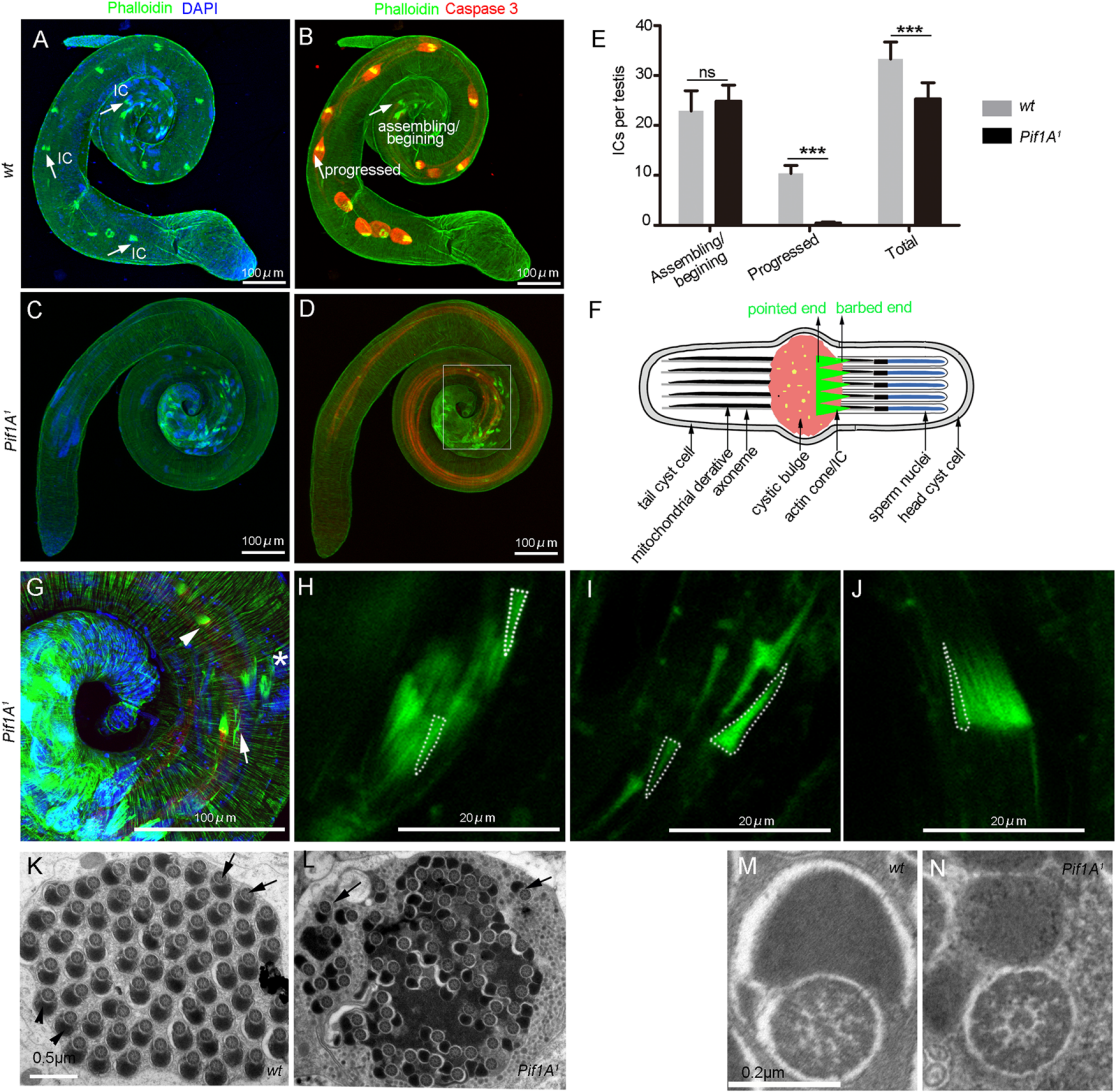
Mutation of *Pif1A* results in abnormal individualization complexes. **a**, **b** Moving individualization complexes (ICs, marked with phalloidin, green) were seen along the sperm tails in the testis of wild-type animal (**a**, arrows), while there was almost no moving IC in the testis of *Pif1A*^*1*^ mutants (**c**, **d**). **e** Quantitation of ICs. The number of ICs that were just assembling or begin to move in *Pif1A*^*1*^ testes was similar to that in the control animals, but the numbers actively progressing as well as the total number of ICs in the *Pif1A*^*1*^ testes (11 testes that were scored) were obviously reduced compared to these of wild-type (10 testes that were scored). *P*-values were determined by a Student’s *t-*test and indicate the statistical significance of the difference and were as follows: *P < 0.05, **P < 0.01, ***P < 0.001. **f** Schematic diagram of spermatid individualization. The top of the long triangle like actin cones always orientates towards the nuclei in wild-type testes. **g** The enlargement of the panel in **d**. The higher magnification of the star, arrow, arrowhead in **g** were **h**, **i**, **j**, respectively. **h**–**j** In *Pif1A*^*1*^testis, actin cone synchronization was disrupted. **h** In *Pif1A*^*1*^ testis, the ICs started moving and the actin cones were not synchronized. **i** In *Pif1A*^*1*^ testis, scattered actin cones moved only a short distance away from the nuclei. Some of the barbed ends of actin cones were orientated away from the nuclei rather than towards the nuclei as in the wild-type; **j** The actin cones seemed to be moving together but the barbed ends of actin cones were all orientated away from the nuclei. **k** Each wild-type cyst contained 64 pairs of spermatid axonemes (arrows) and major mitochondria derivatives (arrowheads) enclosed by a plasma membrane. **l** Individualization defects in the *Pif1A*^*1*^ mutant; the cyst contained only a few individualized regions (arrows). Most of the axoneme/mitochondrial pairs were not separated by plasma membrane and cytoplasm/membranous organelles were still present. The axoneme of the *Pif1A*^*1*^ mutants appeared normal but the major mitochondria derivative structure looked smaller compared to wild-type. Immunostaining of the squashed wild-type testes. **m** magnified TEM figures of the axonemal microtubule organization of wild-type animals. **n** magnified TEM figures of the axonemal microtubule organization of *Pif1A*^*1*^ mutants

We next investigated the spermatid individualization process by visualizing the F-actin component of IC with fluorescent-conjugated phalloidin staining. In WT testes, the IC was correctly assembled around the nuclei and traveled down the cysts (Fig. 5a and Movie S1), and the WBs were evident (Fig. 5b, Movie S3). However, in the *Pif1A*^1^ mutants, the IC movement was limited and disorganized (Fig. 5c, d and Movie S2), where only a few actin cones moved a short distance from the nuclei (Fig. 5e, h). The orientation of the actin cones had been reversed in *Pif1A*^*1*^ mutant (Fig. 5i, j), with no moving CB in the cyst (Movie S4). The barbed end of the actin cones remains close to the nuclei in the WT testis^39–41^ (Fig. 5f), whereas, in *Pif1A*^*1*^ testes, the barbed end presented on the reverse side from the nuclei (Fig. 5i, j). Similar ICs formed at the beginning and almost zero progressed uniformly, in which the total number of ICs per testis was significantly reduced in the mutants (Fig. 5e).

We also examined the cyst during the individualization process using transmission electron microscopy (TEM). In the WT, very little cytoplasm was left in individualized sperm and that each axoneme/mitochondria pair was tightly surrounded by a clear integral plasma membrane (Fig. 5k) and the axonemal microtubules showed a 9 + 2 organization (Fig. 5m). However, in the *Pif1A*^*1*^ mutants, only a few axoneme/mitochondria pairs occurred, and many sperm tails were enclosed within the same membrane and surrounded by a large amount of cytoplasm (Fig. 5l), although the organization of axonemal microtubules in *Pif1A*^*1*^ mutant spermatids seemed normal (Fig. 5n). The nebenkern of *Pif1A*^*1*^ is smaller than that of the WT (Fig. 5m, n and Fig. S3B). The mRNA levels of the related nuclear genes^42–48^ indicated that none but merlin, that has been previously demonstrated to be important for the formation and modification of the nebenkern^47,48^, was significantly downregulated (Fig. S3E). These data together suggest that *Pif1A* might be required for proper aligning and synchronously movement of IC.

### Individualization failure is not caused by a problem in nuclear shaping

**Fig. 6:**
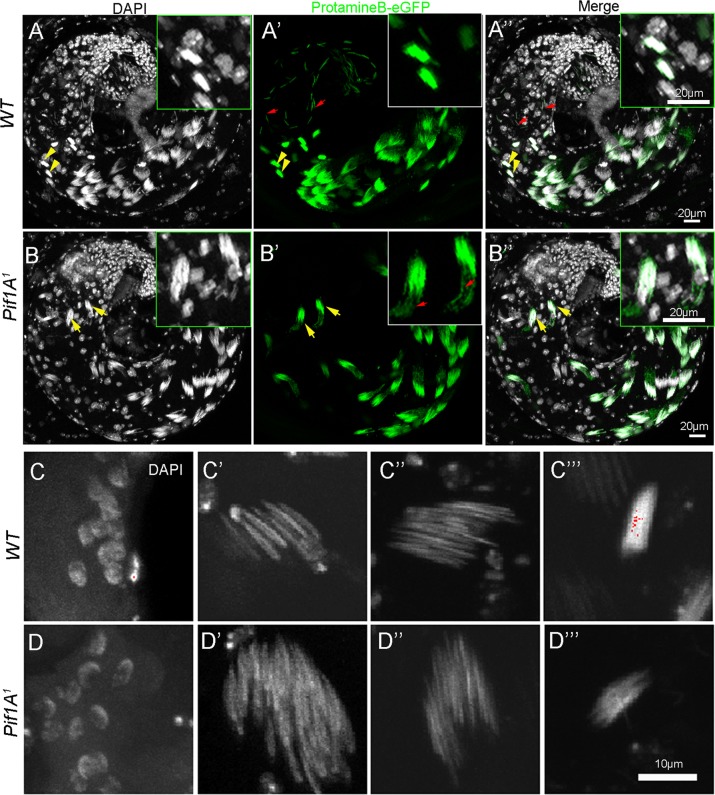
Individualization process is defected in *Pif1A*^*1*^ mutant. **a**, **aʹʹ** and *Pif1A*^*1*^ testes (**b**, **bʹʹ**). (**a**, **b**); DAPI, (**aʹ**, **bʹ**); GFP (representing Protamine B). The lower right corner panes in **a**, **aʹ** are enlargement diagrams of the yellow arrowheads and in **b**, **bʹ** are yellow arrows. **c**, **cʹʹʹ** Nuclei of wild-type animals, **d**, **dʹʹʹ** nuclei of *Pif1A*^*1*^ mutants. **c**, **d** Young elongating nuclei. **cʹ**, **dʹ** Early canoe stage nuclei. **cʹʹ**, **dʹʹ** Late canoe stage nuclei. **cʹʹʹ** Nuclei after individualization. **d**ʹʹʹ Late canoe stage nuclei

During nuclear condensation, somatic histones had been replaced by protamines. In *Drosophila*, the mammalian homolog of the protamines and transition proteins were identified as ProtamineA/Protamine B and Tpl94D^49,50^. We constructed ProtB-eGFP in *Pif1A*^*1*^ mutants background and evaluated the GFP patterns. ProtB-eGFP did not express in young elongating nuclei and early canoe stage spermatids but begun expression from the late canoe stage spermatids onwards^50^. The GFP signals in late canoe stage spermatids were detected in both *Pif1A*^*1*^ and WT animals (Fig. 6aʹ, bʹ). After individualization, the ProtB-eGFP became more condensed and corresponded to and overlapped the nuclei (Fig. 6aʹ). GFP signals were clearly detectable in mature sperm in the testis lumen in WT (Fig. 6aʹ, aʹʹ). In *Pif1A*^*1*^ mutants, the intensive ProtB-eGFP overlapping the needle-shaped nuclei were less defined (Fig. 6bʹ), indicating they had begun to be progressively eliminated.

Spermatid nuclei serve as a physical scaffolding for the assembly of the individualization complex^18,48^. We checked the nuclear shaping during spermiogenesis. In morphology, we could observe the round nuclei, canoe-shaped nuclei and typical needle-shaped nucleus in the testes of both WT animals and *Pif1A*^*1*^ (Fig. 6c, dʹʹ). The transcription levels of the nuclear shaping related genes^37,51–55^ remained unaltered in *Pif1A*^*1*^ (Fig. S4A). However, comparing to that in the WT, fully compacted nuclei are tightened after removing unneeded cytoplasmic components (Fig. 6a, aʹʹ, c, cʹʹ), the needle-like nuclei in *Pif1A*^*1*^ are toward to lose (Fig. 6b–dʹʹʹ). Immunostaining with Caspase 3 in the cyst assembly towards to the terminal epithelium region, where the mature sperms are released into the testis lumen and then pass into the seminal vesicle, the signal was not detectable in the WT (Fig. S6A), but obvious in *Pif1A*^*1*^ mutants (Fig. S6B). This indicated that up-regulated Caspase 3 and cells death occurred at the end of spermatogenesis.

### Lipid metabolism genes are downregulated in the testis in absence of Pif1A

**Fig. 7:**
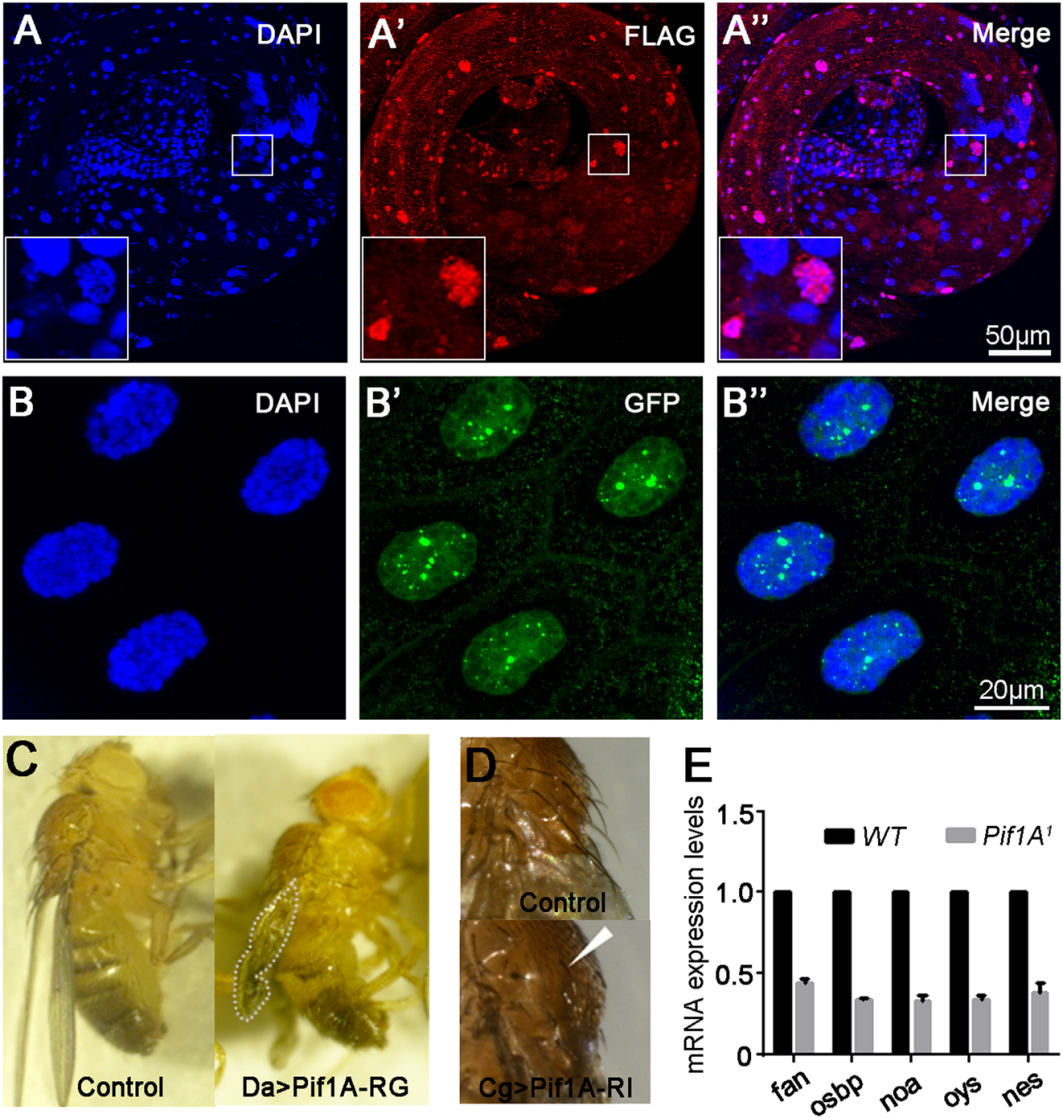
Pif1A influence lipid metabolism genes. **a**,-**aʹʹ** The expression of Flag-tagged Pif1A-RI in the testis from BL42670 animals (**a**ʹ). Nuclei were labeled with DAPI (**a**) and merged (**a**ʹʹ). **b**,-**bʹʹ** The expression of GFP-tagged Pif1A-RG in salivary glands (ppl>GFP-FifaA-RG) (**b**ʹ). Nuclei were labeled with DAPI (**b**) and merged (**b**ʹʹ). **c** Wing expansions after eclosion. White dots indicate the unexpanded wings of ovexpressing Pif1A-RG animals. **d** Dorsal bristles after eclosion. The white triangle indicates shorter bristles. **e** RT–PCR amplification of lipid metabolic genes from testes of wild-type (WT) and *Pif1A* mutants. rp49 was used as a loading control

The observations of irregular pattern and reduced number of ICs in *Pif1A*^*1*^ are similar to phenotypes caused by downregulation of myosin VI^25,28,56^. We sought to analyze the transcription levels of myosin VI, that acts in a structural capacity in the IC^25^, and cortactin and Arp2/3 complex, which play critical roles in regulating actin polymerization^22,27,57^. Real-time PCR results revealed no significant changes in transcription levels of actin-related genes in the testes of *Pif1A*^*1*^ and WT (Fig. S4B). However, when we examined expression levels of a group of lipid metabolic genes, the mRNA levels of *nes, oys, noa, osbp* and *fan* were dramatically downregulated in the testes of *Pif1A*^*1*^ mutants (Fig. 7e).

We finally examined the cellular localization of Pif1A protein by fusion proteins generated in our experiments. Both eGFP-Pif1A-RG and Pif1A-RI-Flag were detected mainly in the nucleus (Fig. 7a, bʹʹ). Immunostaining with Eya antibody and Pif1A-RI-Flag correspondingly evidenced its spermatid-specific expression (Fig. S2). We tested Pif1A overexpression phenotype and observed that adult flies with overexpression of Pif1A-RG driven by Daughterless-Gal4 displayed unexpanded wings and that adult flies with overexpression of Pif1A-RI driven by a hs-Gal4 displayed abnormal dorsal bristles (Fig. 7c, d). For Pifl1A, both deletion and overexpression mutants resulted in similar phenotypes to those of OSBP, respectively, where OSBP had also been demonstrated genetically and physically to regulate the individualization process^30^. These results suggest that Pif1A might act upstream of lipid metabolism genes, such as OSBP, to regulate individualization during *Drosophila* spermatogenesis.

## Discussion

In this study, we have applied *Drosophila* tool kit with the UAS/GAL4 system and CRISPR/Cas9 methods to determine gene function in specific aspects of spermatogenesis, linking abnormal gene function with infertility. Pif1A, the homolog of human CCDC157 that has been shown to be downregulated in men with idiopathic NOA, has been confirmed here to be required for spermatid individualization in a *Drosophila* model. *Pif1A*^*1*^ resulted in compromised male fertility but left female fertility unaffected, which is agreed to Miller’s communication about the mutation Pif1AZ3–5009 (Miller, K. 2017.3.9, www.flybase.org). We confirmed that loss of Pif1A neither effect on germ cell division nor spermatid elongation, but sperm maturation.

In *Drosophila*, the *Pif1A* gene has five annotated transcripts, however, only the *Pif1A-RG* and *Pif1A-RI* are highly expressed in the testes of WT animals with *Pif1A-RH* and *Pif1A-RJ* being barely expressed (Fig. S5). The *Drosophila* sperm proteome analysis also predicted the *Pif1A-RG* transcript of *Pif1A* to exist in sperm^58^. Overexpressing Pif1A-RG and/or Pif1A-RI transgenes driven by a ubiquitously expressed tubulin-gal4 or daughterless-gal4 in the *Pif1A*^*1*^ mutants was not able to rescue the male infertility. One possible explanation is that the overexpression of Pif1A-RG and/or Pif1A-RI, as driven by tubulin-gal4 or daughterless gal4 in *Pif1A*^*1*^ mutant background, does not specifically display during spermatogenesis. The *Pif1A* mutants were frame shift mutants, and the duplication of the *Pif1A* genome (Pif1A-GFP.FPTB) could partially restore the male reproductive capacity (Fig. 1c). Our consideration raised for this partial rescue is that the insertion position of Pif1A-GFP.FPTB might not be strong enough to produce sufficient Pif1A protein.

Using the *Pif1A* mutant testis as a spermatogenesis model system to uncover aspects of male infertility, we were able to assess gene function by screening through the whole spermatogenic developmental processes from GSC division to spermatid differentiation, including the spermatid nucleus shaping and the spermatid elongation processes. Without Pif1A, disruption of the IC’s synchronic movement occurs where the mutant lacks any CB and WB and displays disorganized actin cones (Fig. 5h–j). The elongated spermatids in the mutant seemed eventually undergoing cell death (Fig. S6).

Lipids are essential membrane structural components^59^. Metabolic regulators of lipids such as *nes, oys, noa, osbp*, and *fan* have been reported to play critical roles, particularly during *Drosophila* sperm individualization^29^. We detected significantly decrease in transcriptional expression of these genes in the *Pif1A* mutant testes. The phenotype of the Pif1A mutant with defects in individualization and male-sterile was highly reminiscent of the *osbp* mutants^30^. These findings suggest that *Pif1A* might act as a potent regulator to control the lipid metabolism, therefore having an effect on plasma membrane remodeling during sperm individualization.

The nuclear localization of Pif1A in the testis (Fig. 7a, aʹʹ) might invite speculation for its roles in cyst cells during spermatogenesis. Cyst cells in flies are analogous to mammalian sertoli cells that provide nutrients and physical support for spermatids and also secrete key regulators involved in spermatogenic development^17,60^. Disruption of such a process is expected to impair spermatogenesis and result in no sperm in the seminal vesicle. In humans, nearly 7% of men from the worldwide general population are infertile. The majority of defects in sperm production are categorized as NOA^61,62^. Three types of NOA patients all show down-regulation of CCDC157^13^(Fig. 2d). The coiled-coil domains contained in CCDC157 have been reported as common oligomerization domains for a wide range of proteins such as structural proteins, motor proteins and transcription factors^63^. None of these molecular functions has been specifically confirmed for CCDC157. Our data reveals that *Pif1A*, the homolog of CCDC157, plays a critical role in the individualization process and thus is essential for male fertility. Our study may inform the potential roles of CCDC157 in human spermatogenesis and may provide a new target for the treatment of human male infertility.

## Materials and methods

### Drosophila stocks and genetics

Flies were reared on standard cornmeal medium at 25 ℃. The following strains were used: w^1118^, tubublin-Gal4, daughterless-Gal4,Cg-Gal4, ppl-Gal4,β2tubulin-GFP; Bloomington *Drosophila* Stock Center Df(3R)BSC478/TM6(BL24982), Dj-GFP (BL5417), Pif1A-GFP.FPTB/Cyo(BL42670), dj-GFP(BL5417), protamine B-eGFP/Cyo(BL58406); bam-Gal4 and tj-Gal4 (kindly provided by Chao Tong); nos-Gal4 and UAS-actin.GFP/CyO; Sb/TM6B (Tsinghua Fly Center).

To generate the *Pif1A* mutants, we employed the “CRISPR/Cas9 method”, the gRNA was designed to recognize a 19-nt target sequence and act to direct Cas9-mediated cleavage of both DNA strands within the target site.

To generate UAS-Pif1A-RI, UAS-Pif1A-RG, and UAS-eGFP-Pif1A-RG transgenenic flies, we amplified the full length *Pif1A* cDNA with the primers below and cloned it into the pUAST-attb vector. These constructs were then transformed into VK33 embryos using the standard P-element-mediated transgenesis protocol.

Pif1A- RG-FCTGCGGCCGCGGCTCGAGATGGCTGAAAACCAAACCAAAACG

Pif1A- RG-RTCACAAAGATCCTCTAGATCAGAGCCGGGCATTCTCGGACGG

Pif1A- RI-FCTGCGGCCGCGGCTCGAGATGGGCAACGAGGAATCCT

Pif1A- RI-RTCACAAAGATCCTCTAGACTATTTCTTAGCTCTGAACAAG

eGFP-Pif1A- RG-F TTCGTTAACAGATCTGCATGGTGAGCAAGGGCGAGGA

eGFP-Pif1A- RG-R ATCTCGAGCCGCGGCCGCCACTTGTACAGCTCGTCCATG

### Fertility test

One-day-old males (1 day) from different genotypes were arranged to mate with wild-type 3–5-day-old virgin females in small cages (each cage containing 20 males and 40 females) for one day before calculating the embryo hatch rate. Embryos were then collected on new apple juice agar plates for 4 h and incubated at 25 °C and 45–70% humidity for 24 h. Hatch rates were determined from the proportion of hatched eggs to total eggs (200 each time randomly selected from the plates). Under microscopy, one can easily distinguish the hatched embryos showing shriveled egg shells from the unhatched eggs with the appearance of plump grains of white rice. This experiment was repeated three times.

### Western blotting

Protein extracts from testes were prepared by grinding tissue in lysis buffer (1xRIPA buffer: 50 mM Tris-HCl, pH 8.0, 150 mM NaCl, 1% IGEPAL CA-630, 0.5% sodium deoxycholate, 0.1% SDS) containing the protease inhibitor cocktail (Roche). The lysates were cleared by centrifugation at 14,000 x *g* for 10 min at 4 ℃. Samples were subjected to sodium dodecyl sulfate polyacrylamide gel electrophoresis (SDS/PAGE) and transferred to polyvinylidene fluoride membrane. Membranes were immunoblotted with the primary antibodies and then probed with the secondary antibodies. Rabbit anti-cleaved Caspase 3 (1:100; Cell Signaling) and mouse anti-tubulin (1:1000; Developmental Studies Hybridoma Bank, E7) were used as the primary antibodies. Blots were treated with the ChemiLucent ECL detection reagents (Millipore) and protein bands were visualized using the Chemiluminescence Imaging System (Clinx Science Instruments, Shanghai, China).

### Transmission electron microscopy

Five days after eclosion the testes were dissected from adult males and prepared for electron microscopy using standard protocols. Thin sections were observed and photographed using a Hitachi H-7650 transmission electron microscope.

### Quantitative RT–PCR

Animal experiments were conducted in accordance with the Guidelines for the Care and Use of Laboratory Animals of Zhejiang University. Total RNA from was extracted using RIzol reagent (Invitrogen) from 5-day-old adult testes. The first-strand cDNA was synthesized from 2 μg of total RNA samples using an Invitrogen^®^ First-Strand cDNA synthesis kit. We used Power SYBR Green PCR Master Mix (Applied Biosystems) to conduct RT–PCR with rp49 used as control. Real-time PCR was performed on an ABI7900HT Fast real-time PCR machine. The PCR primers used are provided in below;

rp49-qf GCTAAGCTGTCGCACAAA, rp49-qr TCCGGTGGGCAGCATGTG;

Pif1A-all-qf CAATGCCAGCTCTTGGAACG, Pif1A-all-qr AATCACGACCCTCGAGATC;

pif-RA-qF AAATCAAATGGTGAAATTAC,pif-RA-qR GTTGGGTTATCTTGACATCC;

pif-RI-qF TCTGGACTGTGTCCATAACC, pif-RI-qR ACAAGGTGGAATTGCTCCTC;

pif-RJ-qFAATAATTTCCCCTGTGTTGA, pif-RJ-qR GATTCTCCTCAATAAATTTG;

pif-RH-qF GTTGCCCAAGACTCCTCTTA, pif-RH-qR TCTCCTCAATTATTTGCATT;

pif-RG-qF CTGTTTACATTACAACATCG, pif-RG-qR TCTCCTCAATCATTAGCTCT;

ark-qf GCCTGCAACCGTATCTGAAGCTAA, ark-qr TCTTCAAGTTAAGCCAGAAGATC; dronc-qf GCGTTAGCAAGCTCCGGAATGA, dronc-qr CCTCGGTTGAAACGTGATTGC;

Nessy-qfATTCTGGCAGGTTATCCGGT, Nessy-qrGGACGTGTAGAAATATCCCA;

Oys-qfTTGTCCAAAGAGCCGTCCTG, Oys-qrCTAGGATGCTTTGGAAGTGC;

Noa-qfTCGGCCTAGATTCCAATTGC, Noa-qrGGTGATGTGGTGATACCAGT;

Osbp-qfGTACGTTCAAATCAATCCCT, Osbp-qrCCACTTGACCTCGTTGTCTT;

Fan-qfCTTTGAGGGACCATTCAACC, Fan-qrCTGCATCTTCCAAAACTCCT;

Ccdc157-qf GGAGGAGAACAAGCGGCTCC, Ccdc157-qr GCCTGTCAACCCTGAGAATG;

L17-qf CGGTATAATGGTGGAGTTG; L17-qr ACCCTTAAGTTCAGCGTTACT.

### Microscopy and imaging

The testes of 5-day adult males were dissected in phosphate-buffered saline (PBS). They were then fixed in PBS containing 4% formaldehyde for 15 min at room temperature and washed three times for 15 min each in PBS containing 1% Triton X-100 (PBT) before being blocked for 1 h in PBT containing 5% BSA. The samples were then incubated with primary antibodies (rabbit anti-cleaved Caspase 3, 1:100, Cell Signaling; Mouse anti-ATP synthase complex V subunit alpha antibody, Abcam, 1:1000) overnight at 4 ℃ followed by four 15 min washes in PBT. The samples were incubated with secondary antibodies Alexa Fluor 555 -conjugated (1:500, Molecular Probes) for 1 h at room temperature. Phalloidin staining was performed using Alexa Fluor 488 (Invitrogen) (1:500) for 30 min at room temperature. 4’, 6-diamidino-2-phenylindole (DAPI) (1 g/ml, Sigma, St. Louis, MO, USA) was added to stain the nuclei. Tissues were mounted and viewed. The slides were observed and photographed using an Olympus FV1000 confocal laser scanning microscope and processed using Adobe Photoshop. To quantify the number of ICs in each testis, a confocal Z series of the testes were acquired at a step size of 1.5 mm. For the measure of nebenkern size, images were processed with ImageJ (National Institutes of Health, Bethesda, MD, USA) to measure the relative areas of nebenkern as an indicator of the size and data were analyzed with a Student’s *t*-test. For the white light images of testes, 5-day adult males were dissected in PBS and placed on a glass slide containing a drop of PBS at 25 °C then obtained using a Nikon SMZ1500 microscope.

For live imaging, the testes of 3 day-old adult males from control animals and *Pif1A*^*1*^ mutants were dissected in Schneider’s medium and observed under a Nikon Eclipse 80i fluorescence microscope with a 20 objective lens. Images were acquired at 29.97 frames per second.

### Sequence and statistical analyses

Alignment analysis was performed using https://www.ebi.ac.uk/Tools/msa/clustalo/. Protein structure prediction analysis was performed using SWISS-MODEL (https://swissmodel.expasy.org/) and visualized by UCSF Chimera program. We calculated and generated the plots underlying the differences of CCDC157 mRNA expression levels in nine types of mouse and human tissues respectively, and in 16 patients with NOA compared with four men with normal spermatogenesis by performing the one-way Analysis of variance (ANOVA) by GraphPad Prism 6 (GraphPad Software, Inc., La Jolla, CA, USA). The expression levels of CCDC157 were analyzed by Mann–Whitney test.

### In situ hybridization

Whole-mount adult testis RNA in situ hybridization (ISH) was performed as described^64^. Digoxygenin (dig)-labeled RNA probes are in vitro transcribed from a template synthesized by (RT)-PCR; Pif1A probe was generated by using Pif1A-RG mentioned above as the template with primers Pif1A-F, GGCTACACTTCTTCTATACA; Pif1A-R TACGACTCACTATAGGATTTTGTTCTCCAGCGTG. The antisense probe was generated by using primers Control-F TAATACGACTCACTATAGGCTACACTTCTTCTATACA; Control-R GGATTTTGTTCTCCAGCGTG. The Pif1A probe was the five annotated transcripts common sequence. Detection is through alkaline phosphatase-conjugated anti-dig antibodies followed by a color reaction.

## Supplementary information

Supplementary information

Movie 1

Movie 2 ICs-Pif1A

Movie 3 CB-WT

Movie 4 CB-PIF1A
