# Supplementary material for: Drosophila Pif1A is essential for spermatogenesis and is the homolog of human CCDC157, a gene associated with idiopathic NOA
Source: Cell Death Dis. 2019 Feb 11;10(2):125. doi: 10.1038/s41419-019-1398-3 (PMC6370830; doi:10.1038/s41419-019-1398-3)
Supplement: Supplementary file 1 — Supplementary information [file 41419_2019_1398_MOESM1_ESM.docx]

Supplementary information for

*Drosophila* Pif1A is essential for spermatogenesis and is the homolog of human CCDC157, a gene associated with idiopathic NOA

**Supplement Table 1 Numbers of eggs laid in four hours of each cage**

|  | Average | STDEV | F value | P value |
| --- | --- | --- | --- | --- |
| WT F × WT M | 233 | 18.33 |  |  |
| WT F× *Pif1A* M | 239.67 | 13.20 | 0.683 | 0.636 |
| WT F×*Pif1A*/DF M | 233.33 | 16.50 | 0.895 | 0.982 |
| WT M × *Pif1A* F | 246.33 | 17.47 | 0.952 | 0.413 |
| WT M × *Pif1A*/DF F | 243.33 | 11.24 | 0.547 | 0.452 |

F=females, M=males


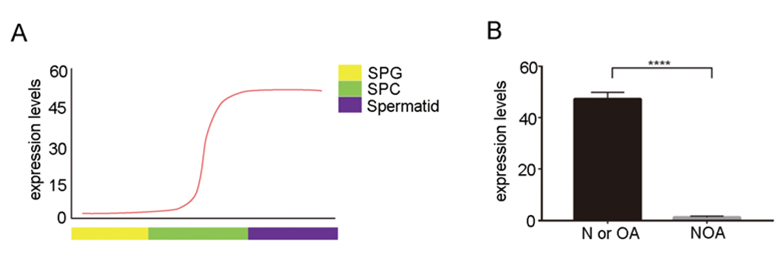


**Supplement Figure 1. A cell-type specific expression pattern of human CCDC157 during spermatogenesis.** Single-cell RNA-sequencing data (NCBI Gene Expression Omnibus (GEO) accession number: GSE106487) underlying a hierarchical model of human testicular cells^1^ were used to evaluate the expression pattern of CCDC157. (A) By dividing ScRNA-seq data of 2854 individual testicular cells from donors with normal spermatogenesis into three clusters according to the rank of the stage-specific marker genes including GFRA1, KIT, STAR8 (spermatogonia stages)^2, 3^, SPO11, OVOL2, NME8 (spermatocyte stages)^4^, and TEX29, NFKBIB, IQCF3, LELP1 (spermatid stages)^1^, we found that CCDC157 is highly expressed throughout spermatid stages. Line chart shows the relative expression levels of CCDC157 during spermatogenesis stages (Fig. S1A). (B) Totally, 2,854 individual testicular cells from donors with normal spermatogenesis showed significant higher expression of CCDC157 than 174 testicular cells from one NOA donor (Fig. S1B). Mann-whitney test, ****, P < 0.0001; ***, P < 0.01; **, P < 0.05. SPG, spermatogonia; SPC, spermatocyte.

_
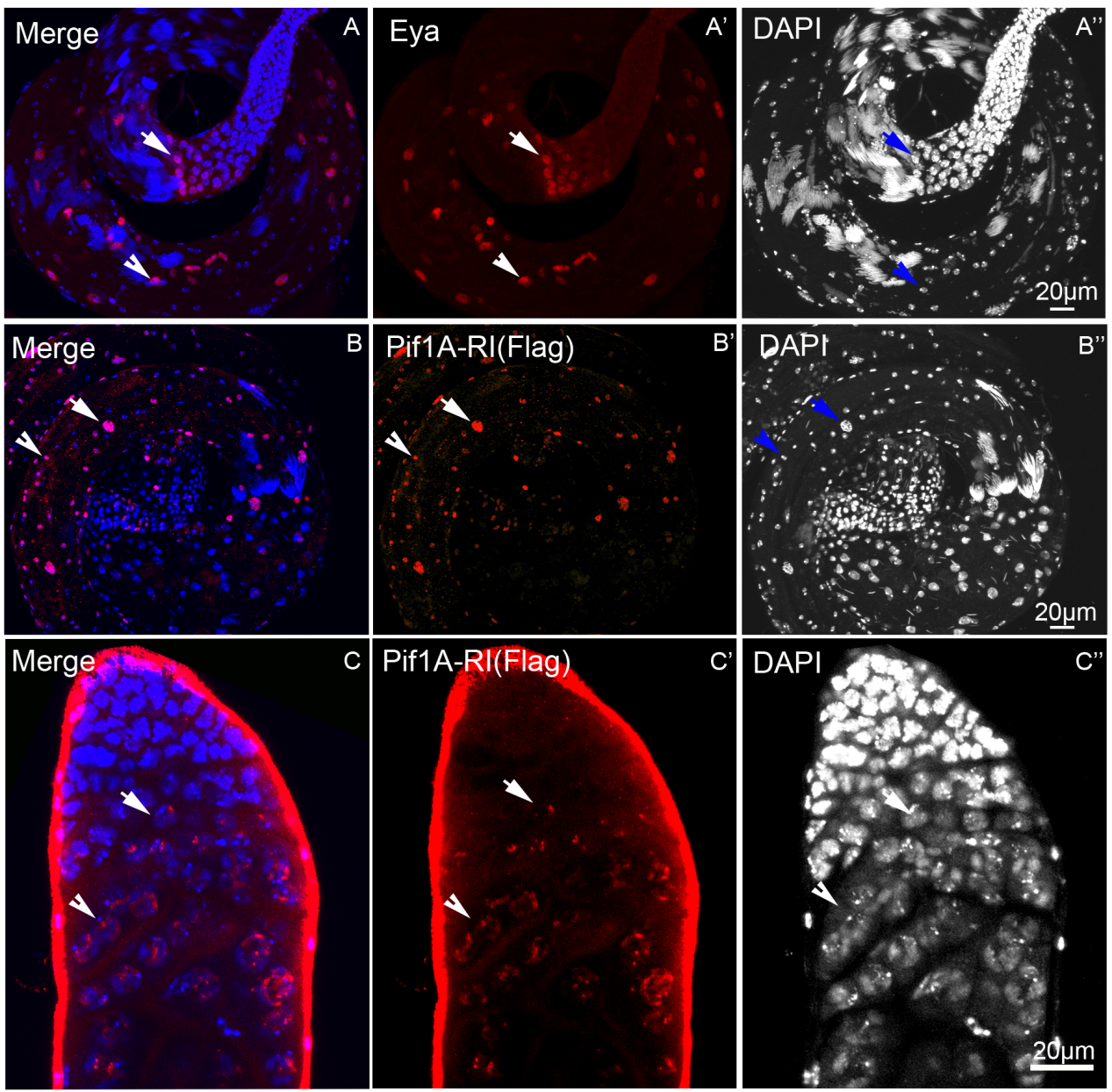
_

**Supplement Figure 2.** An alternative immunostaining with Eya antibody and Pif1A-RI-Flag correspondingly evidenced its spermatid-specific expression. (A-A’’) anti-Eya marks mature cyst cell marker; Arrowhead indicates a nuclear marked by Eya in the middle of the testis and the arrow indicated a nuclear marked by Eya at the terminal epithelium region. (B-B’’) anti-Flag represents Pif1A-RI; Arrowhead indicates a big nuclear and arrow indicates a small nuclear. We can clearly see the types of the nuclei are different, which suggests that Pif1A-RI could not co-localized with Eya. (C-C’’) enlargement diagrams of anti-Flag (representing Pif1A-RI), which showed its expression in the germline nuclei.


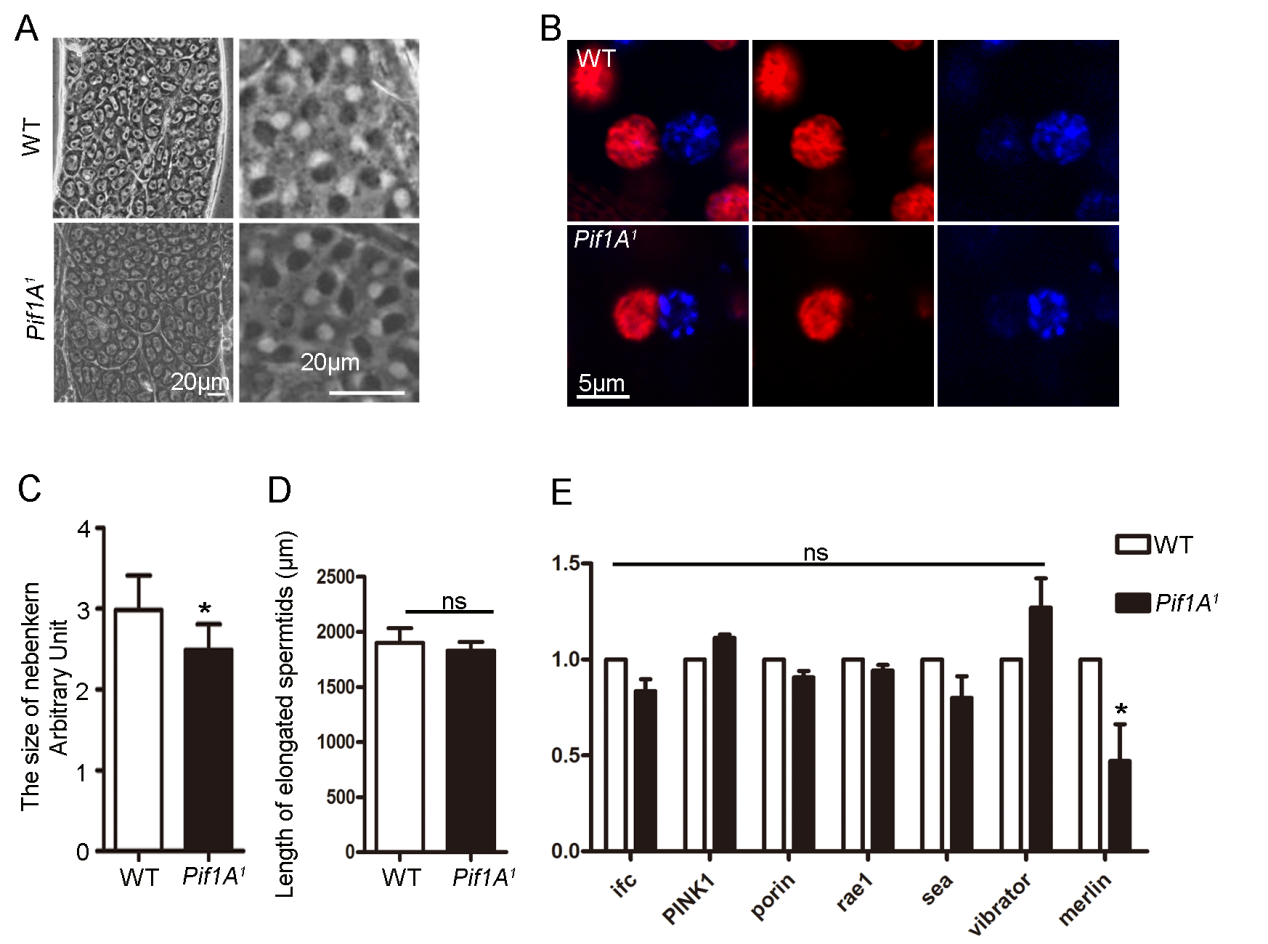


**Supplement Figure 3**.

(A) Comparison of different spermatogenic stages of wild-type and *Pif1A^1^* mutant testes. (Left) Primary spermatocyte cysts at the 16-cell stage. (Right) Onion (64-cell) stage spermatid cysts. (B) Nebenkern morphogenesis stained by ATP5A at the onion stage. (C) the average size of nebenkern in the testes of wild type animals and *Pif1A^1^* mutants and the size of the nebenkern in *Pif1A^1^* mutants is significant smaller than that of the wild type. (D) The length of elongated syncytium of 64 spermatids. There is no obvious difference between the length of elongated spermatids of *Pif1A^1^* mutant and wild type animals. (E) The transcriptional levels of genes related to mitochondrial function or morphogenesis in wild type animals and *Pif1A^1^* mutant.


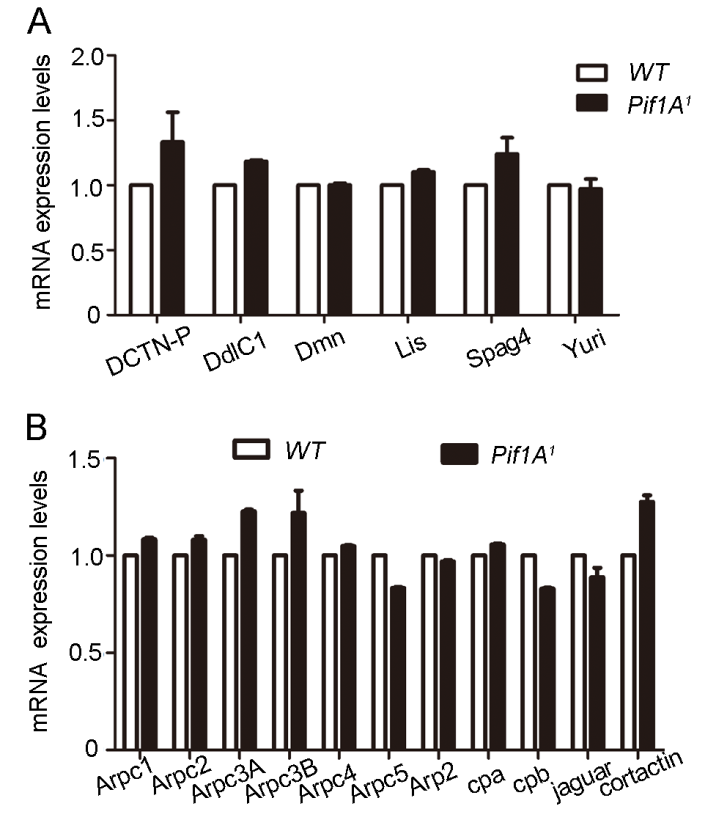


**Supplement** **Figure 4. Two groups of genes transcriptional levels check in *Pif1A^1^* mutant.**

(A) The transcriptional levels of genes related to nuclear shaping have no significant changes between wild type animals and *Pif1A^1^* mutant. (B)The transcriptional levels of the genes related to consisting of actin cone and IC migration have no significant changes between wild type animals and *Pif1A^1^* mutant.


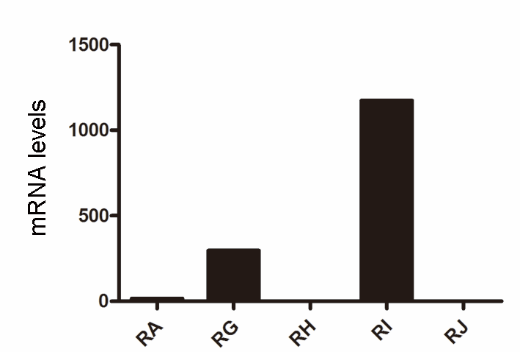


**Supplement Figure 5.**

Real-time PCR analysis of *Pif1A* 5 annotated transcripts in the testes of wild type animals.


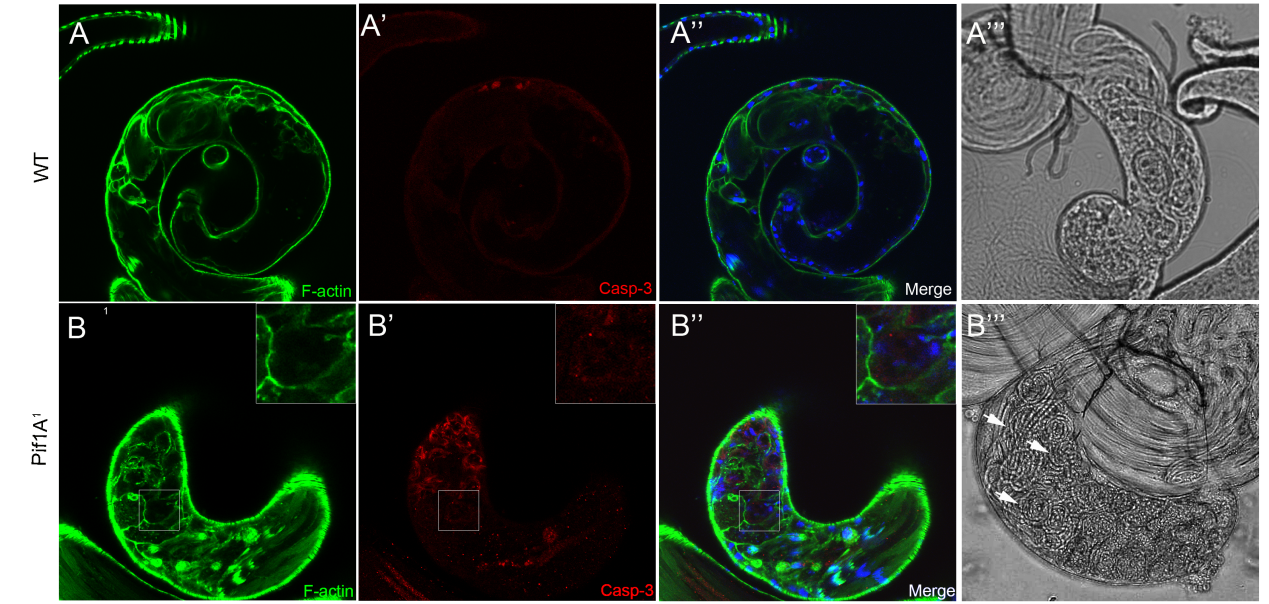


**Supplement Figure 6.**

Caspase 3 staining for the base of the teseis in *Pif1A* mutant (B-B’’) and control animal (A-A’’). No Caspase 3 signal was detected in the entire cyst assembly towards to the terminal epithelium region, where the mature sperms following coiling, are released into the testis lumen and then pass into the seminal vesicle of WT (A’). In the contrast, the Caspase 3 signals were obvious in the cyst of *Pif1A^1^* mutant, indicating cell death occurred towards the end of spermatogenesis in the mutant (B’). Light images of the base of the testes in *Pif1A* mutant (B’’’) and control animal (A’’’), white arrows shows the accumulated of coiled but failed to pass into the seminal vesicle spermatids.

**Primers**

Jaguar-qf GATGACAACTGCGAACTCATGCTG,

Jaguar-qr GCGTATGGCTTTATCCGCAATAG;

cortactin-qf GGCTGGGACCACGTCGAAA,

cortactin-qr CTTGGAGTAATCCTTCTGAGAC;

Arpc1-qf GGATCGAACCCAGATTGCCTTATC,

Arpc1-qrGCGTCCACACATAGGCATTAC;

Arpc2-qf GACACGGAAGAAGGCTACAATGTT,

Arpc2-qr GTAGTTAATGACGGCACGCTT;

Arpc3A-qf TACGAAATCAAGTCCGACGTGGAT,

Arpc3A-qr CGCATCAGATCCGCATCCTG;

Arpc3B-qf CGAAGTTAAGTCGGAGGTGGAC,

Arpc3B-qr CAGCGGAAATCCCGCATCTC;

Arpc4-qf GGTGGACTTTGTCATTAGCTTC, Arpc4-qr GTTGCCTTCACCTAGAACCGT;

Arpc5-qf GAATGTGAAGGACCACGCCCT, Arpc5-qr CTTCTCATGCCACTGCAGCAG;

Arp2-qf GATGATTGAGGTGATGTTCGAG, Arp2-qr GGCAAACTCCTCGTAGACG;

Arp3-qf GGGAGTACACCGCGGAGATAAT,

Arp3-qr ATTATCTCCGCGGTGTACTCCC;

cpb-qf ATGTCGGAAATGCAGATGGAC, cpb-qr GATCTTCAGCGGCTGGTC;

cpa-qf CAACGATCTGGGAAACGGTCG, cpa-qr GTGTCTATAATGACTGGCAGTG;

Dmn-qf GTGGTGGAAATACTGCCG, Dmn-qr TTCTGCTCAATCTCTGCG;

Lis-qf CCAGCATAACGAGGGTAA, Lis- qr ACATCCTGCACAGAGTCC;

Yuri-qf TCCTTAGTCGGCGAGATAG, Yuri-qr CCCTGTTGATTGAGCTGTG;

Ddlc1-qf TTGAAAAGGACATTGCGGC, Ddlc1 qr ACGATCCAAAGTTGCGACC;

Spag4-qf CTCATTTGTGCTGCTCTCA, Spag4-qr GCCACTTTGCTGTCTATCT;

DCTN1-p150-qf TCGCCGACTTGCAGGAACA,

DCTN1-p150 qr CGCAGCACCTCCTTTTTGG;

Ifc-qf: ACACTTTATCTCGGAGCACT, Ifc-qfr: AGCATCGATGGCTTAGGAGG;

Vib-qf: CAACCACCTACCAGTCAAAGA, vib-qr: CTTCGTCTGGTCCTCGATG;

PINK-qf: GATTACCGACAGGACCAAT, PINK-qr: AGCCACTGTAGGATCTCCG;

Merlin-qf: CAAACAATCGACCATCAGCC, Merlin-qr: GATGCGTTAGATGCAATTGG;

Porin-qf: CCTCAGTCTGGAAACAAGA, Porin-qr: CCATCGTTGACAGCTGTGTG;

Rea1-qf: GACAAGACCCTAAAGTTCTG, Rea1-qr: AATGGAAATGGCACGGTGC;

1. Wang M, Liu X, Chang G, Chen Y, An G, Yan L*, et al.* Single-Cell RNA Sequencing Analysis Reveals Sequential Cell Fate Transition during Human Spermatogenesis. *Cell Stem Cell* 2018, **23**(1875-9777 (Electronic))**:** 599-614.

2. von Kopylow K, Staege H, Spiess AN, Schulze W, Will H, Primig M*, et al.* Differential marker protein expression specifies rarefaction zone-containing human Adark spermatogonia. *Reproduction* 2012, **143**(1)**:** 45-57.

3. Endo T, Romer KA, Anderson EL, Baltus AE, de Rooij DG, Page DC. Periodic retinoic acid-STRA8 signaling intersects with periodic germ-cell competencies to regulate spermatogenesis. *Proc Natl Acad Sci U S A* 2015, **112**(18)**:** E2347-2356.

4. Taniguchi H, Katano T, Nishida K, Yao I, Morimoto Y, Matsuda T*, et al.* Expression of hOvol2 in the XY body of human spermatocytes. *Andrologia* 2017, **49**(1).
