# Supplementary figures and images for: Drosophila Pif1A is essential for spermatogenesis and is the homolog of human CCDC157, a gene associated with idiopathic NOA
Source: Cell Death Dis. 2019 Feb 11;10(2):125. doi: 10.1038/s41419-019-1398-3 (PMC6370830; doi:10.1038/s41419-019-1398-3)

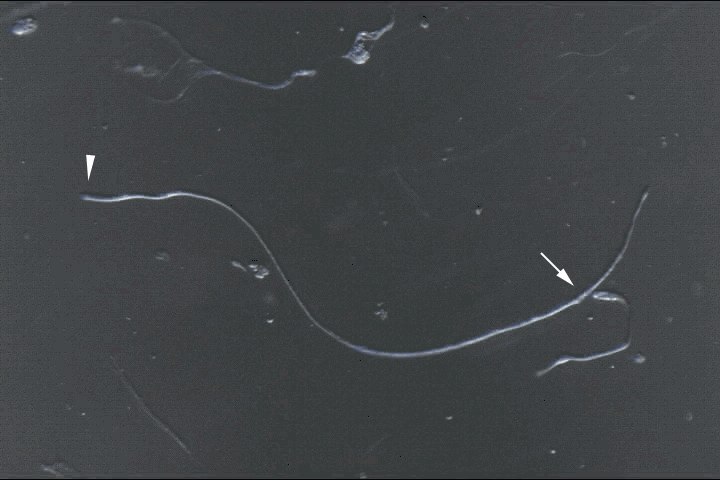

Supplement: Supplementary file 4 — Movie 3 CB-WT [file 41419_2019_1398_MOESM4_ESM.gif]

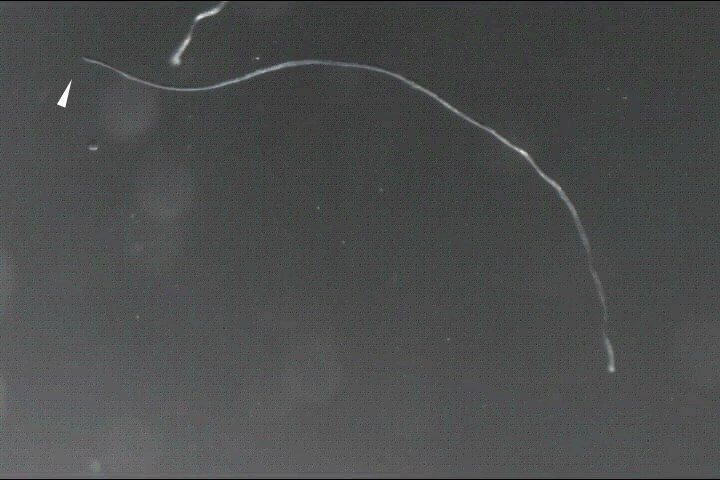

Supplement: Supplementary file 5 — Movie 4 CB-PIF1A [file 41419_2019_1398_MOESM5_ESM.gif]
